# FeOOH Nanosheets Coupled with ZnCdS Nanoparticles for Highly Improved Photocatalytic Degradation of Organic Dyes and Tetracycline in Water

**DOI:** 10.3390/molecules29122913

**Published:** 2024-06-19

**Authors:** Jingren Yang

**Affiliations:** State Environmental Protection Key Laboratory of Environmental Health Impact Assessment of Emerging Contaminants, Shanghai Academy of Environmental Sciences, Shanghai 200233, China; yangjr@saes.sh.cn

**Keywords:** FeOOH nanosheets, ZnCdS nanoparticles, Z-scheme heterojunction, photocatalytic degradation, organic pollutants

## Abstract

Developing a low-cost and highly efficient semiconductor photocatalyst for the decomposition of organic pollutants and antibiotics is highly desirable. Herein, FeOOH nanosheets were prepared using a liquid-phase stirring technique and combined with ZnCdS (ZCS) nanoparticles to construct FeOOH/ZCS nanocomposite photocatalysts. The photocatalytic efficiency of the FeOOH/ZCS nanocomposite was evaluated for the decomposition of various pollutants, including rhodamine B, methylene Blue, and tetracycline. The FeOOH/ZCS nanocomposite exhibited significantly higher photocatalytic performance for the decomposition of various organics. Moreover, the optimized FeOOH/ZCS retained more than 90% of its initial photocatalytic activity even after five successful runs. Radical quenching test and electron spin resonance (ESR) analysis revealed that hydroxyl radicals (^•^OH) play a dominant role for the decomposition of organics. The FeOOH/ZCS Z-scheme heterojunction significantly facilitates higher charge transfer efficiency and the generation of reactive radicals, resulting in excellent photocatalytic degradation performance. This work offers a new approach to synthesis FeOOH-based photocatalyst for the elimination of organics and antibiotics in water.

## 1. Introduction

In recent years, various environmental pollutants such as dyes, antibiotics, and their derivatives have significantly threatened human health [1,2,3]. To overcome these issues, various treating technologies, including photocatalytic degradation [4,5,6], adsorption [7,8], biological treatment [9], and flocculation and sedimentation, have been extensively explored for the elimination of organics in water [4]. Among these approaches, semiconductor photocatalysis has been regarded as an up-and-coming advanced oxidation process (AOPs), which has superior stability, high efficiency, and green sustainability for water treatment [5,6,7,8]. However, several enormous challenges remain in photocatalysis, such as the separation of photocatalysts, low transfer efficiency, and poor redox performance [9,10,11]. Thus, extensive research is needed to develop a highly efficient photocatalyst with high migration efficiency and redox ability. To date, some strategies have been developed to address this issue, including morphological engineering, defects, and doping heteroatoms.

Zinc cadmium sulfide (ZnCdS) solid solutions have recently attracted significant research attention due to their low cadmium content and tunable band structure properties among various metal sulfides [12,13]. This has led to an increased interest in their potential applications in photo(electro)catalysis [14,15]. It is worth noting that the utilization of ZnCdS nanocrystals in the decomposition of different organics and antibiotics in water has been explored in a limited number of studies. However, the pure ZnCdS nanoparticles face challenges such as the small size of construction-related low recovery, its tendency to cluster, and thus, low efficiency and reactivity greatly hinder its practical applications. To overcome these intrinsic challenges, constructing ZnCdS-based nanocomposites that incorporate suitable cocatalysts was regarded as an ideal solution. In recent decades, a number of efficient and inexpensive cocatalysts, such as cobalt [16], nickel [17], and iron-based [18] oxides (hydroxides), have been developed for photocatalytic production or degradation. Among these metals, Fe is the most abundant transition metal and less toxic than Co and Ni. FeOOH has raised much attention among iron-based oxides due to its flexible structure, corrosion resistance, and relative stability [19,20,21]. Ultrathin two-dimensional nanosheets are commonly utilized in various catalytic reactions or as catalyst precursors to endow the large surface area for composite catalysts, leading to the improved photocatalytic performance [22,23,24]. Thus, it is imperative to fabricate hybrid photocatalyst that is both low-cost and efficient via combined FeOOH nanosheets and ZnCdS nanoparticles, which achieve the optimal photocatalytic degradation performance in water treatment.

Herein, we developed a simple synthetic method to obtain ultrathin FeOOH nanosheets modified ZnCdS nanoparticles (noted as FeOOH/ZCS) as a 2D/0D heterojunction photocatalyst for photocatalytic degradation reactions. The morphology and photocatalytic performances of the FeOOH/ZCS nanocomposites were investigated systematically. Through a series of systematic photocatalytic experiments and comprehensive characterization, the prepared FeOOH/ZCS photocatalyst exhibited a much higher photocatalytic performance for degrading rhodamine B (RhB), methylene blue (MB), and tetracycline (TC) compared to FeOOH nanosheets and ZnCdS nanoparticles. Radical quenching and ESR analysis indicated that the target contaminants were degraded via ^•^OH-dominated radical pathway. We further propose a mechanism that significantly enhances the photocatalytic decomposition activities of the FeOOH/ZCS nanocomposite.

## 2. Results

### 2.1. Catalyst Structure

**Figure 1:**
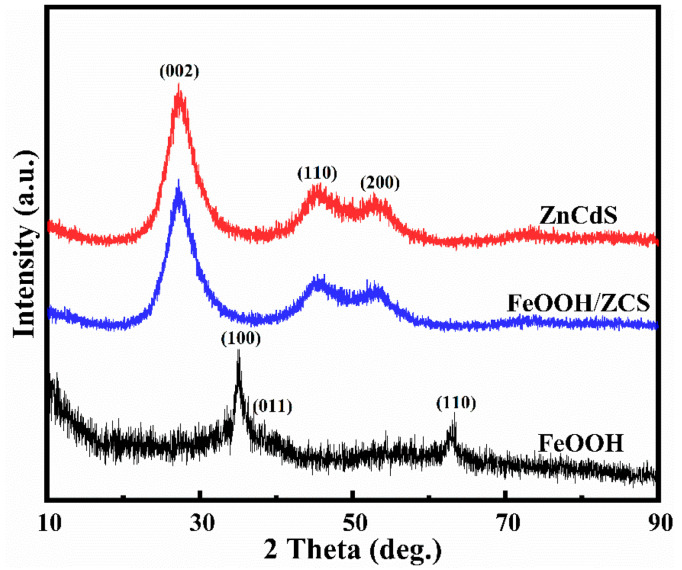
XRD spectra of ZnCdS NCs, FeOOH NSs, and FeOOH/ZCS.

From the XRD spectra in Figure S1, the patterns of ZnCdS can be assigned to the cubic phase of CdS (PDF#10-0454) and ZnS (PDF#05-0566). This observation suggests that the ZnCdS was not physically acquired from a single CdS and ZnS, but rather a solid solution of ZnCdS was formed [25]. Figure 1 illustrates the XRD patterns of ZnCdS, FeOOH, and FeOOH/ZCS samples. The diffraction peaks located at 27.8°, 45.9°, and 53.5° can be credited to (002), (110), and (200) crystal planes of hexagonal ZnCdS (JCPDS No. 89-2943), respectively [26]. The crystallite size of ZnCdS in FeOOH/ZCS and ZCS NPs was calculated to be 1.2 and 1.7 nm by Scherer equation. Three diffraction peaks at 35.5°, 40.4°, and 63.0° correspond to the (100), (011), and (110) planes of hexagonal δ-FeOOH (JCPDS No. 77-0247) [27]. The crystallite size of FeOOH in FeOOH/ZCS and pure FeOOH NSs was calculated to be 3.4 and 3.8 nm by the Scherer equation. Notably, the FeOOH/ZCS exhibit diffraction peaks similar to pristine ZnCdS. This similarity can be attributed to the low content of FeOOH in combined nanocomposite.

**Figure 2:**
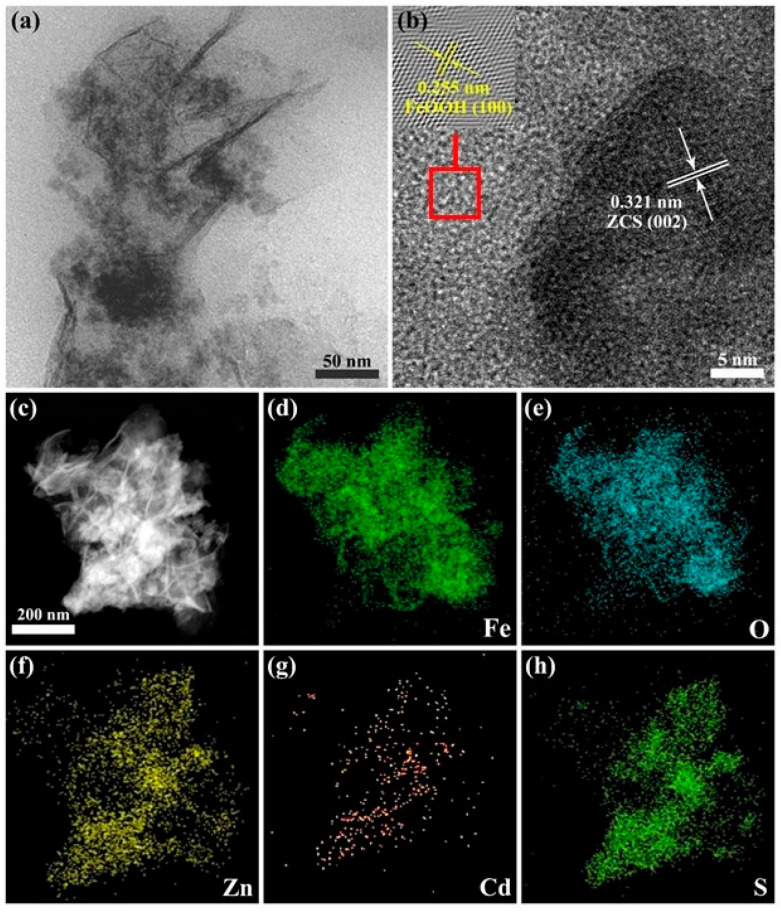
(**a**) TEM, (**b**) HRTEM, (**c**–**h**) HAADF, and elemental mapping images of FeOOH/ZCS heterostructures.

The morphology of the obtained samples was disclosed with SEM and TEM analyses. As illustrated in Figure S2a–c, the SEM and TEM images displayed a sheet-like structure of FeOOH. Furthermore, the HRTEM image (Figure S2d) showed a d-spacing value of 0.255 nm, corresponding to the (100) plane of the FeOOH. The TEM image (Figure 2a) of the FeOOH/ZCS displayed that the ZnCdS NCs were immobilized on the surface of the FeOOH NSs. This good interfacial connection between ZnCdS nanoparticles and FeOOH nanosheets significantly facilitates the separation as well as transfer of photoexcited charges in photocatalytic process. The HRTEM image of the FeOOH/ZCS nanocomposite (Figure 2b) showed lattice stripes with defined spaces of 0.255 nm and 0.321 nm, which can be assigned to the (100) and (002) planes of FeOOH and ZnCdS, respectively. As can be seen from Figure 2c–h, the high-angle annular dark-field (HAADF) image and elemental mapping analysis of FeOOH/ZCS showed that the detected elements were distributed throughout the FeOOH/ZCS nanocomposite, further confirming that ZnCdS nanoparticles were effectively integrated into the layered FeOOH nanosheets. 

UV-Vis absorption measurement is a valuable way to investigate the band structure of semiconductors. It can be seen from Figure S3a pure ZnCdS NCs showed an intense absorption at the visible light region with an absorption edge at 545 nm, and the corresponding band gap energy (Eg) of the sample was assessed to be 2.53 eV. Pure FeOOH NSs also displayed strong absorption with an Eg of approximately 1.67 eV (Figure S3b). Impressively, the light absorption of FeOOH/ZCS was significantly broadened and red-shifted, with an estimated Eg value of 2.50 eV. These results can be attributed to the mixing of FeOOH NSs, which possessed a small band gap, a large absorption coefficient, and powerful absorption. The increased visible-light absorption and lower band gap energy for the composites could effectively facilitate the visible-light photoreactivity in reaction.

**Figure 3:**
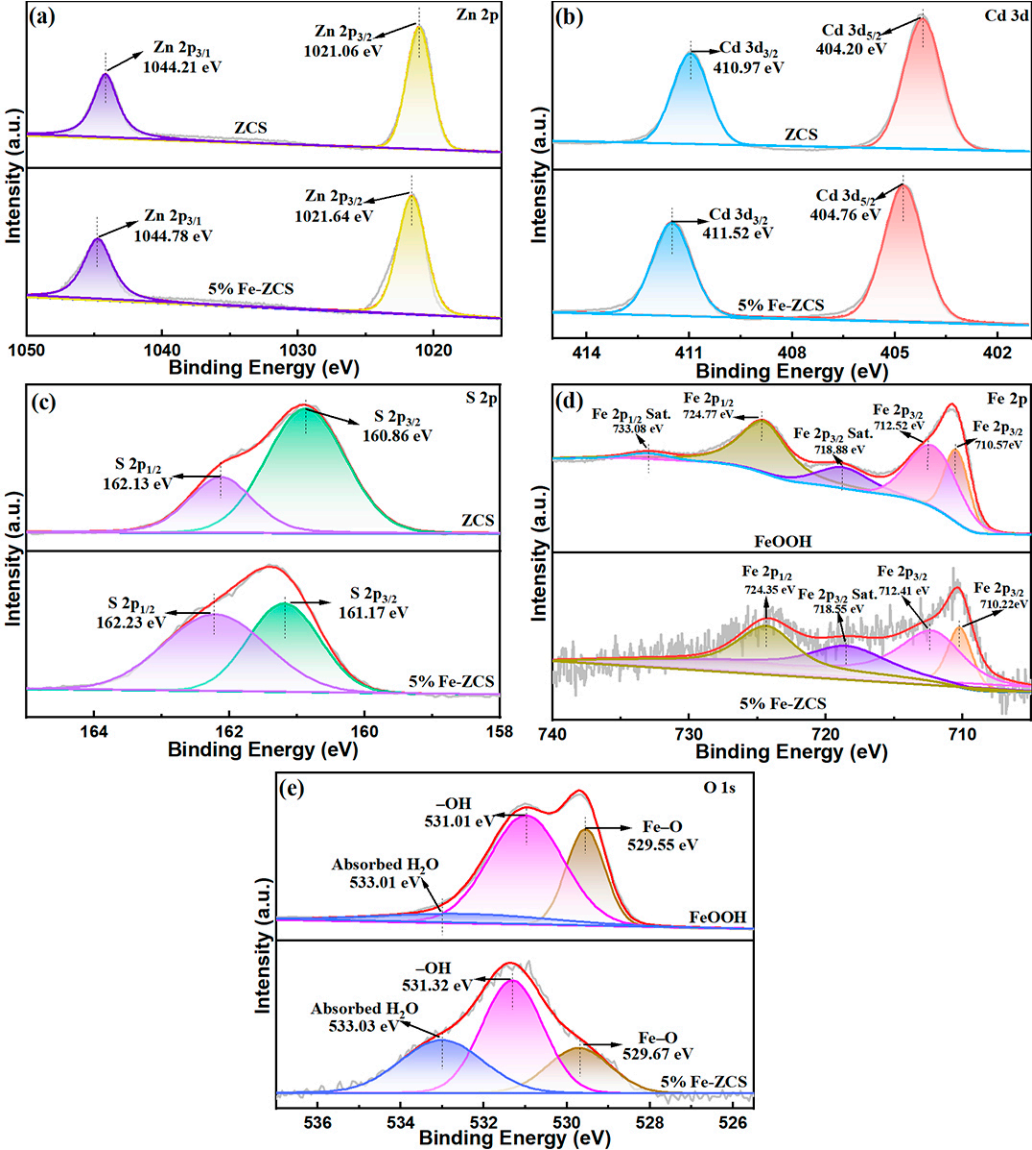
The XPS spectra of ZnCdS NCs and FeOOH/ZCS heterostructures: (**a**) Zn 2p, (**b**) Cd 3d, and (**c**) S 2p. High-resolution spectra of FeOOH NSs and FeOOH/ZCS heterostructures: (**d**) Fe 2p and (**e**) O 1s of the oxygen vacancies.

The chemical composition and electronic states of the obtained ZnCdS NCs, FeOOH NSs, and FeOOH/ZCS heterostructures were studied by XPS. For ZnCdS NCs, Zn 2p at around 1021.1 and 1044.2 eV (Figure 3a), Cd 3d at around 404.2 eV and 411.0 eV (Figure 3b), and S 2p at around 160.9 eV and 162.1 eV (Figure 3c) were proven to be zinc ions (Zn^2+^), cadmium ions (Cd^2+^), and sulfide ions (S^2−^) of ZnCdS NCs, respectively [10,12,28]. For the isolated FeOOH NSs (Figure 3d), two peaks at 712.5 and 724.8 eV can be related to Fe^3+^ ion, and a peak at 710.6 eV is related to Fe^2+^ ion. The existence of Fe^2+^ can be attributed to the formation of oxygen vacancies in FeOOH NSs [20]. The three peaks at 533.0, 531.0, and 529.6 eV in O 1s spectra can be signed by the adsorbed H_2_O, –OH bonds, and Fe–O in FeOOH NSs, respectively (Figure 3e) [29]. It can be seen that the binding energies of Zn 2p, Cd 3d, and S 2p in FeOOH/ZCS were higher than those in ZnCdS NCs, while the binding energy of Fe 2p in FeOOH/ZCS was lower than that in FeOOH NSs, which can be ascribed to the partial electron transfer from ZnCdS NCs to FeOOH NSs [30]. In summary, XPS analysis demonstrated that the deposition of ZnCdS NCs partly covered on the surface of FeOOH NSs, which means that the oxygen vacancies could be a priority deposition site for ZnCdS NCs, thus contributing to the formation of FeOOH/ZCS heterostructures. This result not only intensifies the interfacial contact between the two semiconductors but also facilitates their electronic interactions by providing fast electron transport channels.

### 2.2. Photocatalytic Dye Degradation Performances

**Figure 4:**
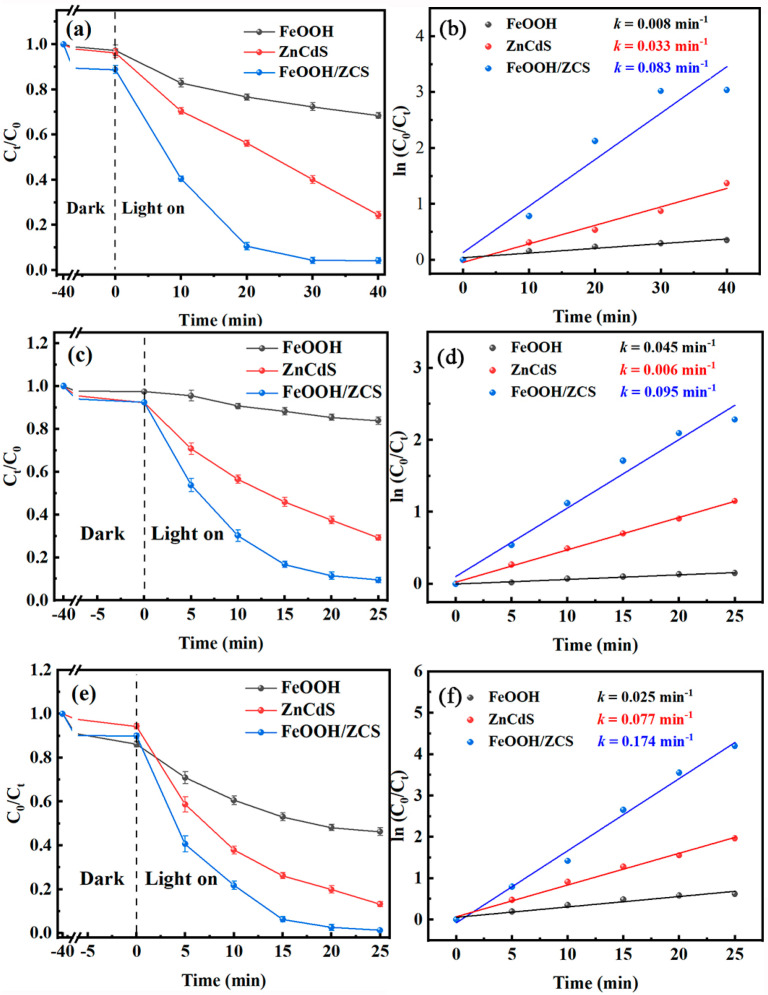
The removal efficiency of as-prepared samples for (**a**) RhB (20 mg/L), (**c**) MB (20 mg/L), and (**e**) TC (50 mg/L) photocatalysis degradation under visible light. First-order kinetic model for (**b**) RhB, (**d**) MB, and (**f**) TC degradation.

In the present work, the photocatalytic activity of the FeOOH/ZCS system was determined for the decomposition of RhB, MB, and TC (Figure 4). The reaction was represented by pseudo-first-order kinetic Equation (1) to further compare the efficiency of photocatalysis.

−ln (*C_t_*/*C*_0_) = *kt* (1)

where C_0_ and C_t_ are the original concentration and the residual concentration, respectively. The photocatalytic performance of ZnS, CdS, and ZnCdS were mainly assessed by the decomposition of RhB. The concentration changes of RhB at 554 nm in the presence of different samples as a function of illumination time during degradation are shown in Figure S4. As shown in Figure S4a, the ZnCdS NCs demonstrated better photocatalytic performance than pure CdS and ZnS for the decomposition of RhB. The 100% RhB removal can be achieved within 60 min using a ZnCdS photocatalyst. In addition, during the photodegradation process, Figure S5 shows that the adsorption intensity significantly decreased as the reaction time increased from 0 to 40 min, indicating the RhB was degraded. Meanwhile, a blue shift in the maximum absorption peak of RhB (at 554 nm) was observed with increased irradiation time, which was ascribed to the de-ethylation reaction during irradiation [31]. To further evaluate the obtained catalyst, the photodegradation experiment of ZnCdS NCs, FeOOH NSs, and FeOOH/ZCS heterostructures for RhB and MB was carried out. As shown in Figure 4a,c, the blank experiment without the photocatalysts demonstrated that the RhB and MB can hardly be degraded. When ZnCdS NCs and FeOOH NSs are applied as photocatalysts, 60% and 30% RhB removal can be achieved after 30 min reaction (Figure 4a). Interestingly, the 98% RhB removal can be achieved within 40 min using FeOOH/ZCS catalyst, which might be attributed to the large contact interface between ZnCdS NCs and FeOOH NSs in FeOOH/ZCS. The large contact interface intensified the photoinduced charge separation and transfer, facilitating the RhB degradation in the FeOOH/ZCS photocatalytic system. 

In addition, the *k* value of FeOOH/ZCS is calculated to be 0.083 min^−1^, higher than that of ZnCdS NCs (0.033 min^−1^) or FeOOH NSs (0.008 min^−1^) (Figure 4b). Meanwhile, 95% MB can be degraded by FeOOH/ZCS after 25 min, which is greater than ZnCdS NCs (72.5%) and FeOOH NSs (18%) under visible light excitation (Figure 4c,d). TC, as the quintessential antibiotic, is widely used in the medical industry and presents a threat to human health. Herein, TC was also employed to further examine the photocatalytic activity of obtained samples (Figure 4e,f). Under visible light excitation, FeOOH/ZCS shows a higher photocatalytic efficiency (99%) than ZnCdS NCs (80%) or FeOOH NSs (50%) after 20 min, which further demonstrated the high photoactivity of FeOOH/ZCS. Compared with reported similar photocatalysts in Table S1, the FeOOH/ZCS exhibited the great potential application in environmental remediation. 

**Figure 5:**
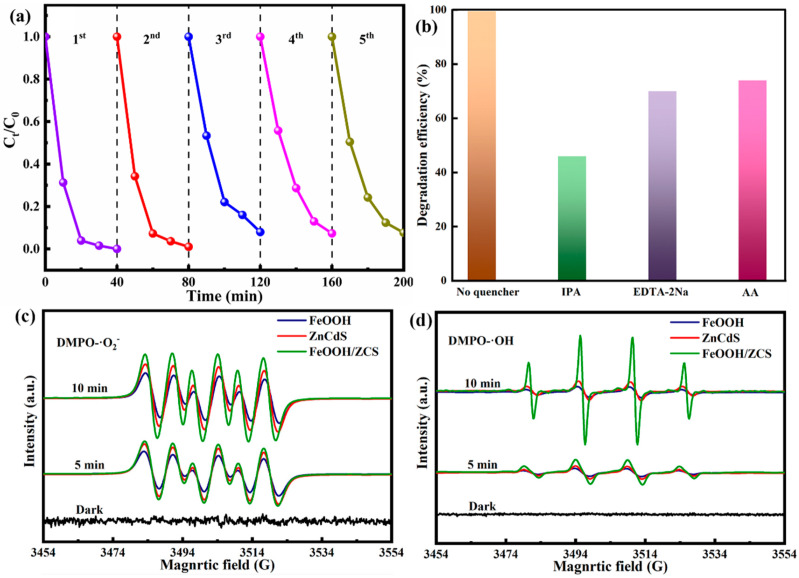
(**a**) Cycling times of the photocatalytic decomposition of RhB using FeOOH/ZCS heterostructures under light conditions, (**b**) degradation efficiency of RhB using FeOOH/ZCS heterostructures with different radical scavengers (reaction conditions: [IPA] = 1 M, [EDTA−2Na] = 1.5 M, [AA] = 1.5 M). EPR spin−trapping measurements of (**c**) DMPO−^•^O_2_^−^ and (**d**) DMPO−^•^OH.

The FeOOH/ZCS photocatalyst has demonstrated superior photoactivity in decomposing various types of organic compounds. To assess the reusability of FeOOH/ZCS, the obtained catalyst reuse test was conducted. As shown in Figure 5a, the FeOOH/ZCS remained almost unchanged photoactivity for the RhB decomposition during the reuse test, implying that it has potential for practical applications. After the succussive cyclic test, the reacted FeOOH/ZCS composite was characterized by XRD to evaluate the integrity of its phase structure. It can be seen from Figure S6, the diffraction peaks of FeOOH/ZCS remained almost unchanged after the cycle run, demonstrating the impressive stability of FeOOH/ZCS. This result indicated that FeOOH NSs are crucial in enhancing the photocatalytic performance of ZnCdS NCs under visible light.

To identify the photodegradation regime driven by visible light, the free radical trapping tests were performed. It is generally known that isopropyl alcohol (IPA), ascorbic acid (AA), and disodium ethylenediaminetetraacetic acid (EDTA-2Na) are effective scavengers for ^•^OH, **^•^**O_2_^−^, and h^+^, respectively. As shown in Figure 5b, AA inhibited the RhB degradation slightly, suggesting that **^•^**O_2_^−^ partially contributes to RhB decomposition. After adding EDTA-2Na, the degradation rate of RhB decreased to 75%, implying that h^+^ can further induce inhibition of RhB degradation. Intriguingly, IPA induced remarkable inhibition on the RhB degradation, revealing that **^•^**OH is the primary active species in the RhB decomposition. In addition, EPR analysis was performed to further validate the generation of free radical during reaction. It can be seen from Figure 5c, no signal appeared under dark condition. Impressively, upon exposure to visible light, a gradual enhancement in the signal intensity corresponding to DMPO–radical **^•^**O_2_^−^ was observed. As shown in Figure 5d, no characteristic peak of DMPO–radical **^•^**OH was detected under dark conditions, but during visible light excitation, an EPR signal with an intensity ratio of 1:2:2:1 for DMPO–radical ^•^OH was observed [32]. Moreover, the intensity of the DMPO–radical ^•^OH signal increased with the irradiation time, indicating more ^•^OH was generated as the photocatalytic reaction. Impressively, compared with the pure ZnCdS NCs and FeOOH NSs, the FeOOH/ZCS composite system showed the strongest characteristic in the ESR test, suggesting that the FeOOH/ZCS hybrid system generated more ROS during photodegradation. The phenomenon was consistent with the photodegradation performance tests.

### 2.3. Proposed Photocatalytic Mechanism

**Figure 6:**
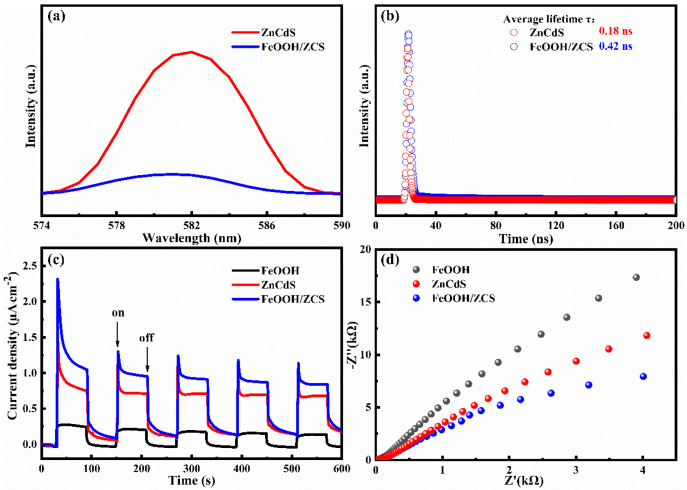
(**a**) Photoluminescence (PL) and (**b**) TRPL spectra of ZnCdS NCs and FeOOH/ZCS heterostructures, (**c**) photocurrent curves, and (**d**) EIS plots of ZnCdS NCs, FeOOH NSs, and FeOOH/ZCS heterostructures.

To further examine the photocatalytic activity of the FeOOH/ZCS, the PL spectra were collected. Typically, the separation rate of photogenerated electrons and holes diminishes with increased PL emission intensity [33]. Figure 6a shows the PL spectra of obtained samples documented at an excitation wavelength of 290 nm at room temperature. Pure ZnCdS NCs exhibited intense luminescence, substantiating the light-induced electron/hole pair production and the next recombination process. For FeOOH/ZCS, the PL intensities decreased significantly, showing that the photogenerated carrier recombination was effectively suppressed by the p-n heterojunction between ZnCdS NCs and FeOOH NSs. In addition, time-resolved PL spectra were employed to estimate the quantum lifetimes of the photogenerated carriers. As displayed in Figure 6b, the average lifetime (τaverage) of FeOOH/ZCS was 0.42 ns, which was comparatively better than ZnCdS NCs (0.18 ns). The extended lifetime reflects increased carrier transfer efficiency and reduced recombination, which is highly favorable for photoactivity [34]. As displayed in Figure 6c, the pure ZnCdS NCs and FeOOH NSs exhibited a weak photocurrent response. Nevertheless, the FeOOH/ZCS composite exhibited a significantly improved photocurrent density, then remained stable after five on/off cycles, suggesting that the formed heterojunction between ZnCdS NCs and FeOOH NSs played crucial role in facilitating charge carrier transfer and inhibiting the recombination of photoinduced electron/hole pairs [35]. It is well accepted that the radius of the arc in the EIS Nyquist diagram mirrors the reaction rate at the electrode surface. Typically, a smaller radius corresponds to a lower electron transfer resistance and therefore means a stronger charge transfer and separation efficiency [12,36]. As illustrated in Figure 6d, FeOOH/ZCS possesses the smallest arc radius than the other pure catalysts, manifesting that FeOOH/ZCS has smaller charge transfer resistance, stronger separation efficiency of photoinduced electron/hole pairs and quicker interfacial charge transfer than pristine FeOOH NSs and ZnCdS NCs, which is in excellent concordance with the observations from PC measurements and photocatalytic performance.

Mott–Schottky (MS) plots of ZnCdS NCs and FeOOH NSs are demonstrated in Figure S7 to illuminate their electronic band structures. The flat-band potential can be derived from the x-axis intercept and is near the Fermi level (EF) [37]. The flat-band potentials were determined from the intercepts on the cross-section of the extrapolation line. It is noted that the flat-band potentials of ZnCdS NCs and FeOOH NSs were −0.41 V and 2.83 V, respectively. Since E_VB_ = Eg + E_CB_, the valence band (E_VB_) potential of ZnCdS NCs and the conduction band (E_CB_) potential of FeOOH NSs were about 2.12 eV and 1.16 eV, respectively.

**Figure 7:**
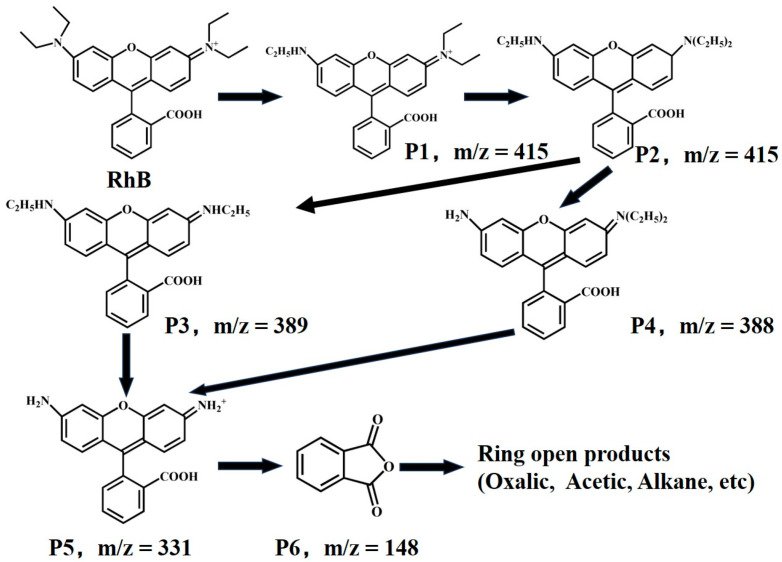
Proposed RhB degradation pathway.

The TOC value of 82.61 mg/L decreased to 11.17 mg/L, which the 86.5% TOC removal was achieved using FeOOH/ZCS system after reaction, indicating RhB was partially mineralized. To identify the main decomposition intermediates of RhB, the LC-MS was performed. As shown in Figure S8, six decomposition intermediates were detected. Then, the possible RhB degradation pathway was proposed. As shown in Figure 7, RhB was attacked by ^•^OH and transformed into P1. Then, the e^+^ was transferred from catalyst to RhB and formed P2. The P2 was further attacked by ROS, leading to the formation of P3, P4, P5, and P6. Finally, the intermediates of P6 was further transformed into ring-opening product, which was ultimately mineralized into CO_2_ and H_2_O.

## 3. Discussion

**Figure 8:**
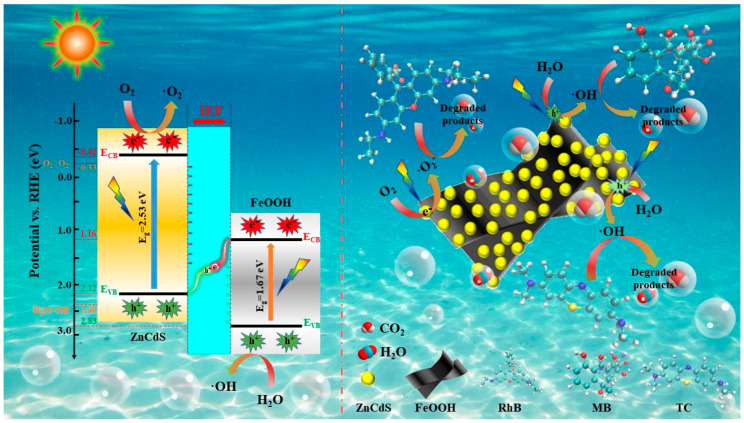
Proposed photocatalytic degradation of FeOOH/ZCS heterostructures.

On the basis of the above analysis, we proposed a potential mechanism for pollutant degradation using FeOOH/ZCS composite photocatalyst under visible light. According to the conventional type II charge transfer mechanism, the electrons in the conduction band of ZnCdS NCs will move to the conduction band of FeOOH NSs, while the holes will be transferred from the valence band of FeOOH NSs to the valence band of ZnCdS NCs. Then, photogenerated carriers can be effectively detached. However, the CB of FeOOH (1.16 eV) is higher than that of O_2_/^•^O_2_^−^ (−0.33 eV), and the VB of ZnCdS (2.12 eV) is smaller than that of H_2_O/**^•^**OH (2.40 eV), indicating that the photoexcited hole cannot theoretically oxidize H_2_O to **^•^**OH. Meanwhile, **^•^**O_2_^−^ cannot be produced during the catalytic reaction [38]. However, free-radical trapping experiments and EPR spin capture techniques revealed that ^•^OH and ^•^O_2_^−^ are produced during the reaction. Therefore, the conventional photoinduced carriers transferring and separation mode does not accommodate this potential scheme. Hence, we should consider another possible mechanism. As shown in Figure 8, ZnCdS and FeOOH are highly activated under visible light excitation to produce e**^−^**-h**^+^** pairs. Benefiting from the formation of heterojunction between ZnCdS NCs and FeOOH NSs, the electrons in the FeOOH NCs CB are easily migrated into the ZnCdS NCs VB, resulting in that ZnCdS NCs CB generated abundant ^•^O_2_^−^. Meanwhile, FeOOH NSs VB produced a significant amount of **^•^**OH due to its stronger oxidation capacity. This result facilitated the effective separation and accumulation of electrons of ZnCdS NCs and FeOOH NSs, leading to a remarkable enhancement in the photocatalytic performance of FeOOH/ZCS composites [39]. Consequently, the formed h^+^, ^•^OH, and ^•^O_2_^−^ exhibited strong oxidation capacity for the decomposition of various organics.

## 4. Materials and Methods

### 4.1. Materials

RhB (98%, Aladdin, Shanghai, China), MB (98%, Innochem, Beijing, China), TC (96%, Aladdin, Shanghai, China), IPA (99%, Innochem, Beijing, China), AA (AR, Fisher, Shanghai, China), and EDTA-2Na (99%, Innochem, Beijing, Shanghai) were directly used.

### 4.2. Synthesis

**Scheme 1:**
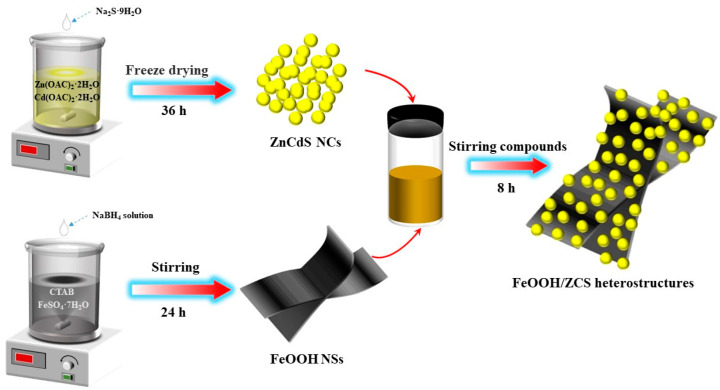
Fabricating scheme of FeOOH/ZCS nanocomposites.

ZnCdS nanoparticles, FeOOH nanosheets, and FeOOH/ZCS heterostructures were synthesized and presented in Scheme 1.

#### 4.2.1. Synthesis of ZnCdS Nanoparticles (Named ZnCdS NCs)

Quantities of 3 mmol of Zn(OAC)_2_·2H_2_O and 3 mmol of Cd(OAC)_2_·2H_2_O were dispersed in 50 mL water. Subsequently, a solution of Na_2_S·9H_2_O (6 mmol, 15 mL) was slowly added under stirring and kept for 8 h. The resulting yellowish precipitates were collected by centrifugation, washed with deionized (DI) water, and processed in a freeze dryer for 36 h. For comparison, pure ZnS and CdS were fabricated by the same way but adding Cd(OAC)_2_·2H_2_O and Zn(OAC)_2_·2H_2_O, respectively.

#### 4.2.2. Synthesis of FeOOH Nanosheets

FeOOH nanosheets (FeOOH NSs) were prepared based on a procedure described in a previous report [40]. Typically, 41.7 mg of FeSO_4_·7H_2_O and 182 mg of CTAB were dispersed in 50 mL of DI water while continuously stirred. Then, 2 mL of a 0.4 M solution of NaBH_4_ was added, and the mixture was stirred for 24 h. The resulting precipitate was collected, washed several times with DI water and methanol, and dried at 45 °C for 24 h.

#### 4.2.3. Synthesis of FeOOH/ZCS Nanocomposites

The FeOOH/ZCS nanocomposites were prepared by a solution-phase method: 95 mg of ZnCdS and 5 mg of FeOOH were dispersed in 15 mL of ethanol and subjected to ultrasonication for 15 min. The resulting mixture was then stirred magnetically for 8 h. Afterward, the nanocomposites were vacuumed and dried overnight.

### 4.3. Characterization 

The morphology of the obtained sample was tested with field emission scanning electron microscope (SEM, Hitachi SU8220, Tokyo, Japan) and transmission electron microscopy (TEM, FEI TECNAI G2 F30, Hillsboro, FL, USA). The structure and compositions of the samples were identified using an X-ray powder diffractometer (XRD, Rigaku, D/MAX 2500V, Beijing, China) and X-ray photoelectron spectroscopy (XPS, Specs, ESCALAB 250Xi, Shanghai, China). The ultraviolet–visible diffuse reflectance spectra were detected by UV-vis spectrophotometer (UV, Shimadzu, UV-3600 Plus, Kyoto, Janpan). The photoluminescence (PL) and time-resolved PL (TRPL) were collected on a transient fluorescence spectrometer (HORIBA, FL3C-111 TCSPC, Paris, France). Photocurrent responses and electrochemical impedance spectroscopy (EIS) plots were performed on a VSP-300 multi-channel potentiostat (Bio-Logic, Cecine Parisse, France).

### 4.4. Photocatalytic Tests

The RhB, MB, and TC concentration were determined by light absorption at 554 nm, 665 nm, and 358 nm under visible light, respectively. The light source was a 300 W Xe lamp (CEL-HXF300, Aulight, Beijing, China) with a 400 nm UV cut-off filter. In a typical RhB degradation reaction, 20 mg of photocatalysts were added to 80 mL of 20 mg/L of RhB. The mixture was stirred for 40 min until adsorption–desorption equilibrium was achieved. The calculated 2 mL of suspension was measured using UV-Vis spectrophotometry during reactions. To test the durability of FeOOH/ZCS, the sample was collected by centrifugation after each cycle and used for photocatalytic RhB dye degradation for 5 cycles. The photocatalytic degradation measurements of MB and TC were similar to those of RhB.

## 5. Conclusions

In summary, 0D ZnCdS nanoparticles deposited on 2D FeOOH nanosheets by liquid-phase precipitation successfully produced FeOOH/ZCS heterojunctions. XPS analysis, EPR tests, and MS provided strong evidence to confirm that FeOOH/ZCS heterojunctions are Z-scheme. The large specific surface area provided abundant active site, absorbed more reactant molecules, and formed Z-scheme heterojunctions that simultaneously block photogenerated electron/hole complexes and promote photocatalytic carrier transfer to generate more reactive radicals and holes, thus enhancing the photocatalytic activity of the FeOOH/ZCS. Furthermore, FeOOH/ZCS maintained its photocatalytic properties, crystal structure, and microstructure after five cycles of RhB photodegradation, indicating that the obtained FeOOH/ZCS heterojunctions possessed superior electrical conductivity and chemical stability. Thus, this work provides a relatively convenient, energy-efficient, and promising strategy for the rational design of efficient and stable Z-Scheme heterojunction photocatalysts for photocatalytic wastewater remediation.

## Data Availability

The data presented in this study are available on request from the corresponding author.

## Supplementary Materials

The following supporting information can be downloaded at: https://www.mdpi.com/article/10.3390/molecules29122913/s1. References [41,42,43,44,45,46,47,48,49] are cited in the Supplementary Materials.
