# Supplementary material for: FeOOH Nanosheets Coupled with ZnCdS Nanoparticles for Highly Improved Photocatalytic Degradation of Organic Dyes and Tetracycline in Water
Source: Molecules. 2024 Jun 19;29(12):2913. doi: 10.3390/molecules29122913 (PMC11206779; doi:10.3390/molecules29122913)
Supplement: Supplementary file 1 [file molecules-29-02913-s001.zip › molecules-3037460-supplementary.pdf]

Supporting Information

FeOOH nanosheets coupled with ZnCdS nanoparticles for highly improved photocatalytic degradation of organic dyes and tetracycline in water

Jingren Yang*[a]

[a] State Environmental Protection Key Laboratory of Environmental Health Impact Assessment of Emerging Contaminants, Shanghai Academy of Environmental Sciences, Shanghai 200233

*Corresponding author.

Email: yangjr@saes.sh.cn (J. Yang)

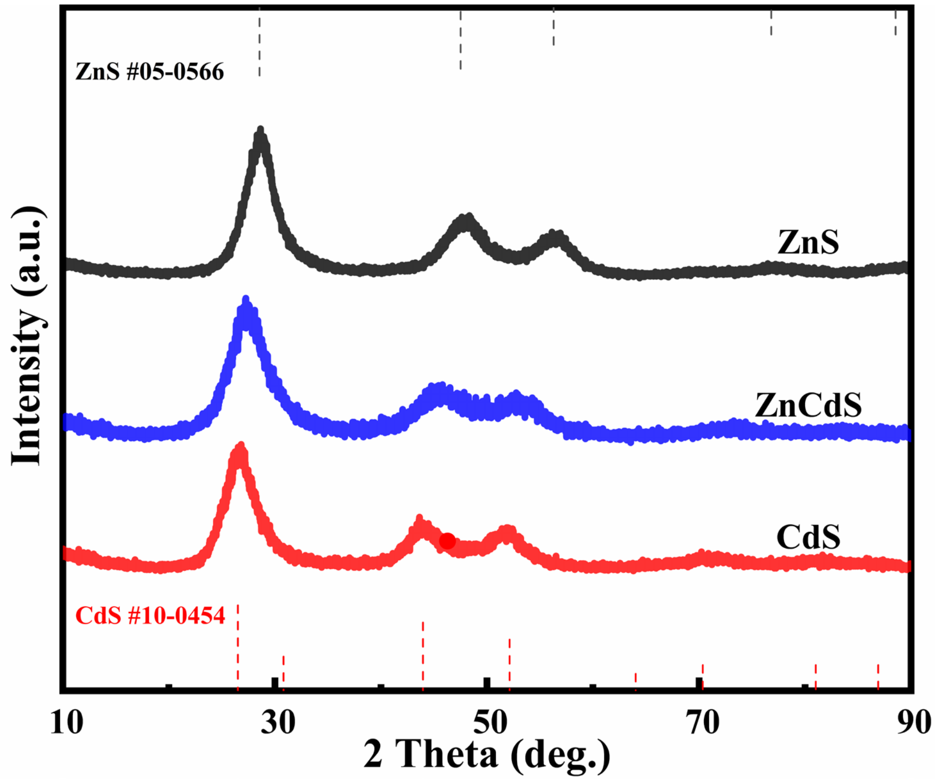

Figure S1. XRD patterns of ZnS, CdS and ZnCdS.

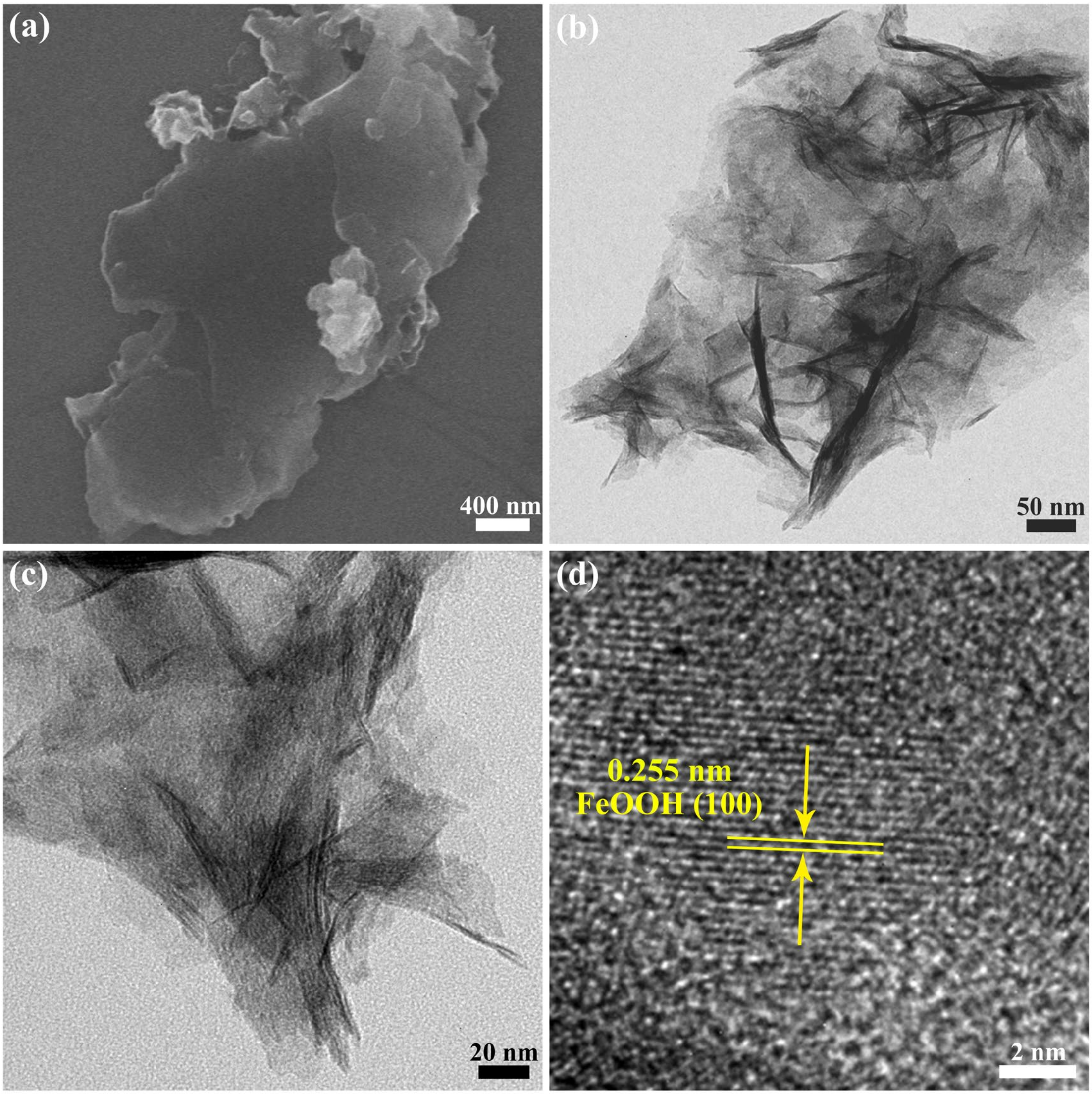

Figure S2. Characterization of FeOOH nanosheets: (a) SEM image, (b,c) TEM images and (d) HRTEM image.

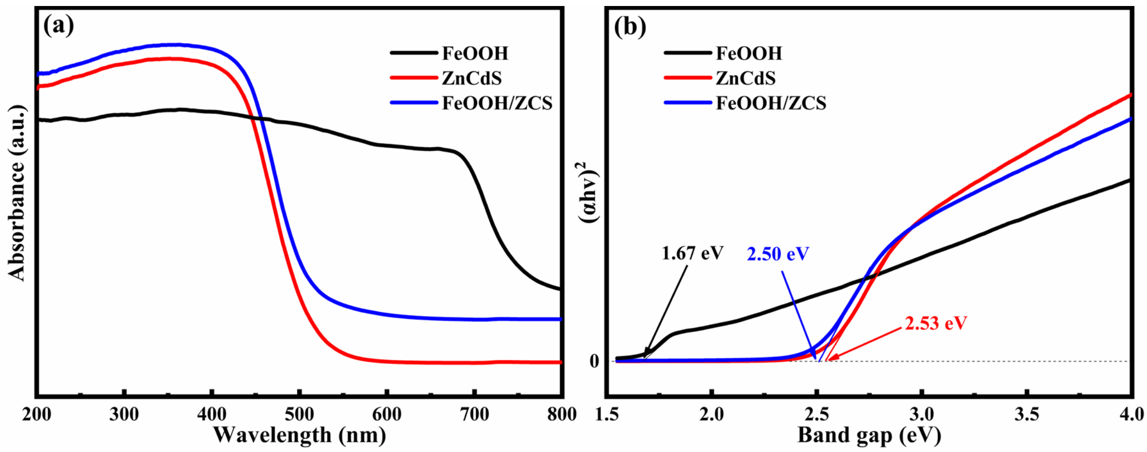

Figure S3. (a) UV–VIS diffuse reflectance spectra (DRS) and (b) the plots of $(\alpha h\nu)^2$ vs photon energy (hv) for the band gap energy of ZnCdS NCs, FeOOH NCs and FeOOH/ZCS heterostructures.

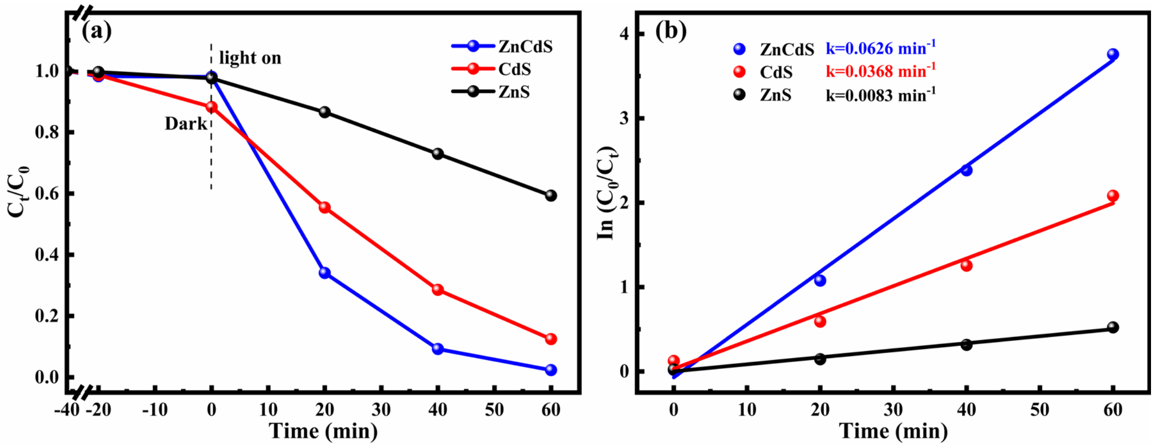

Figure S4. (a) the photocatalytic degradation curve of RhB (20 mg/L) dye in the presence of ZnS, CdS and ZnCdS NCs. (b) the pseudo-first-order Kinetic plots and apparent rate constants of ZnS, CdS and ZnCdS NCs.

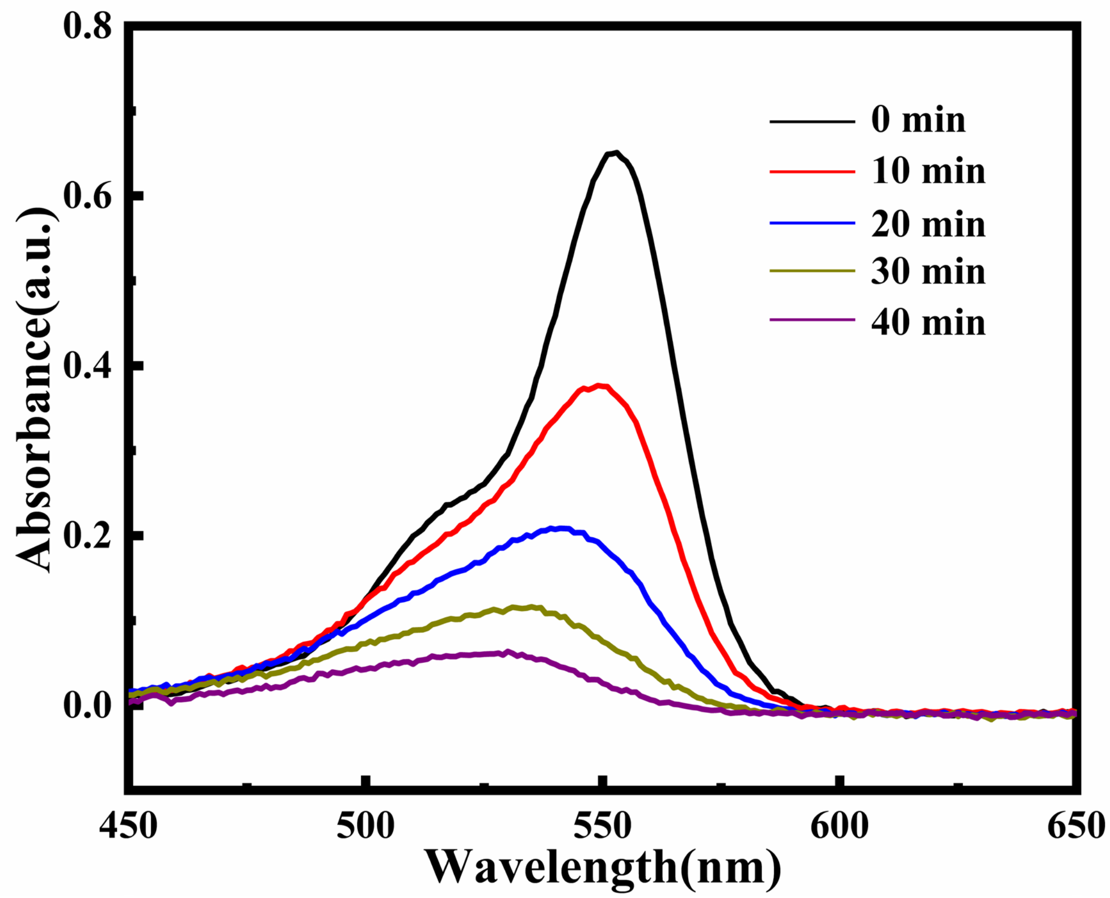

Figure S5. Temporal absorption spectral changes of RhB (20mg/L) during the photodegradation process using ZnCdS NCs.

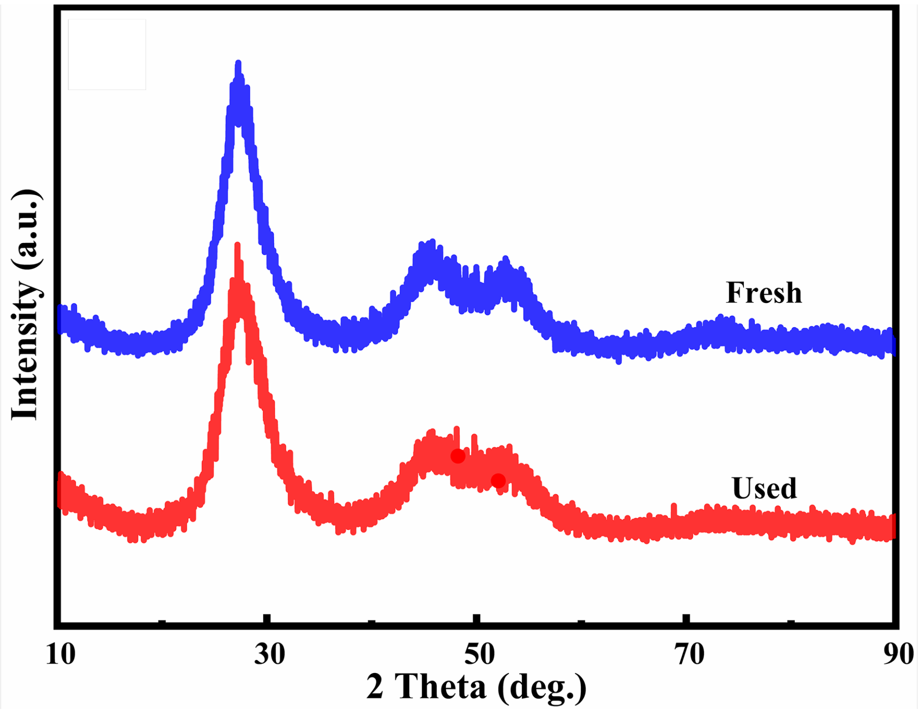

Figure S6. XRD pattern of the FeOOH/ZCS heterostructures after five repeated cycles.

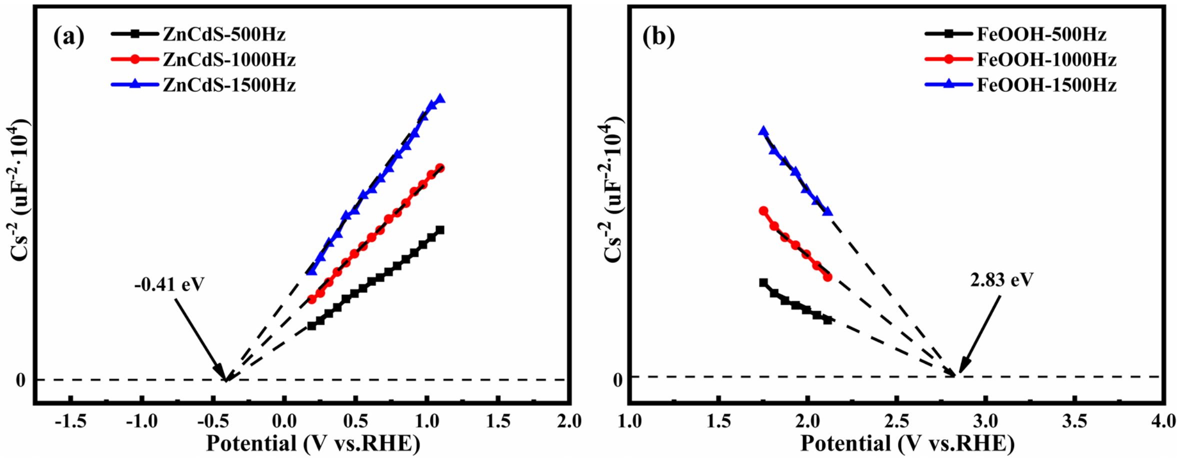

Figure S7. Mott-Schottky plots of (a) ZnCdS NCs. and (d) FeOOH NSs.

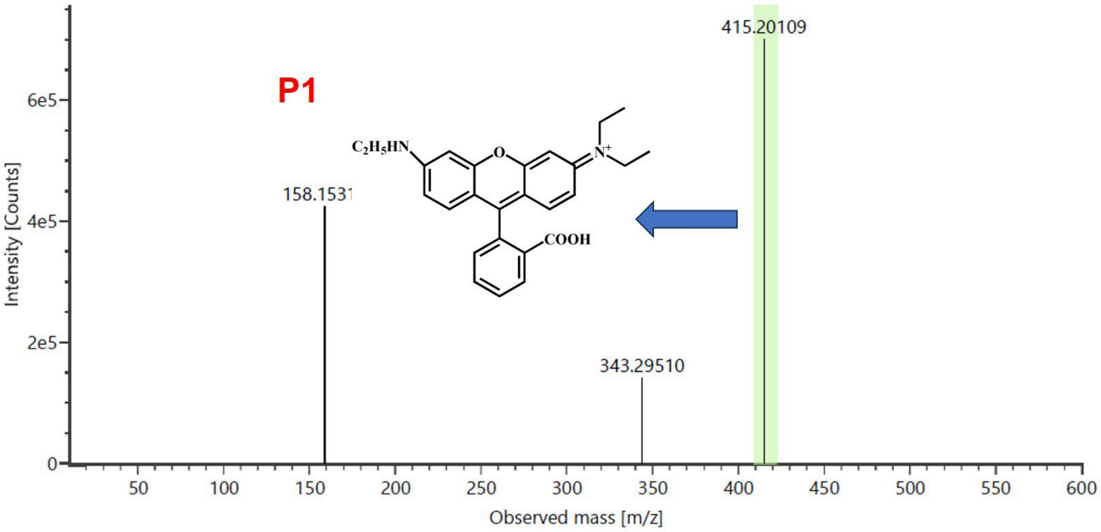
P1
415.20109
158.1531
343.29510
Intensity [Counts]
Observed mass [m/z]
COOH

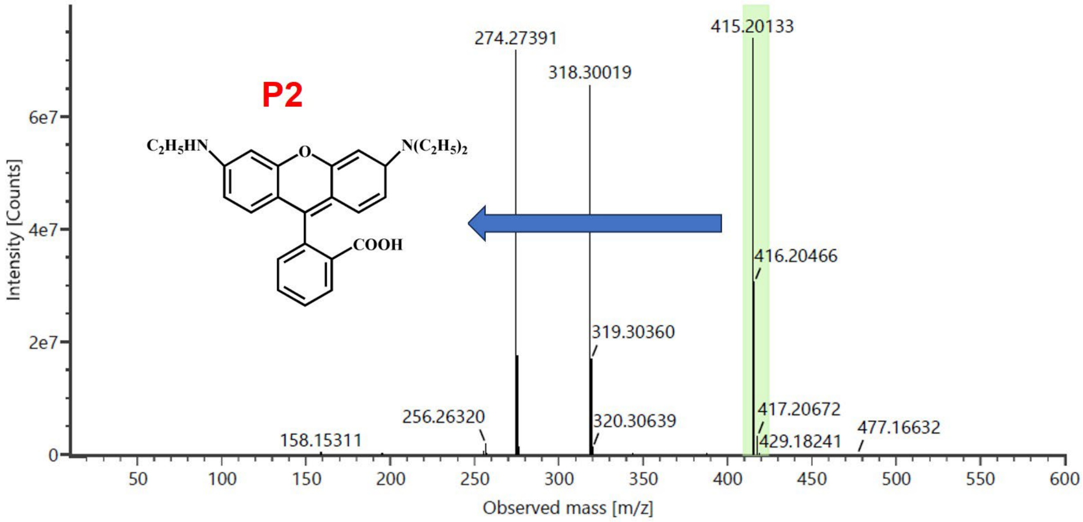
P2
274.27391
318.30019
415.20133
416.20466
319.30360
417.20672
320.30639
256.26320
429.18241
477.16632
158.15311
Intensity [Counts]
Observed mass [m/z]
COOH

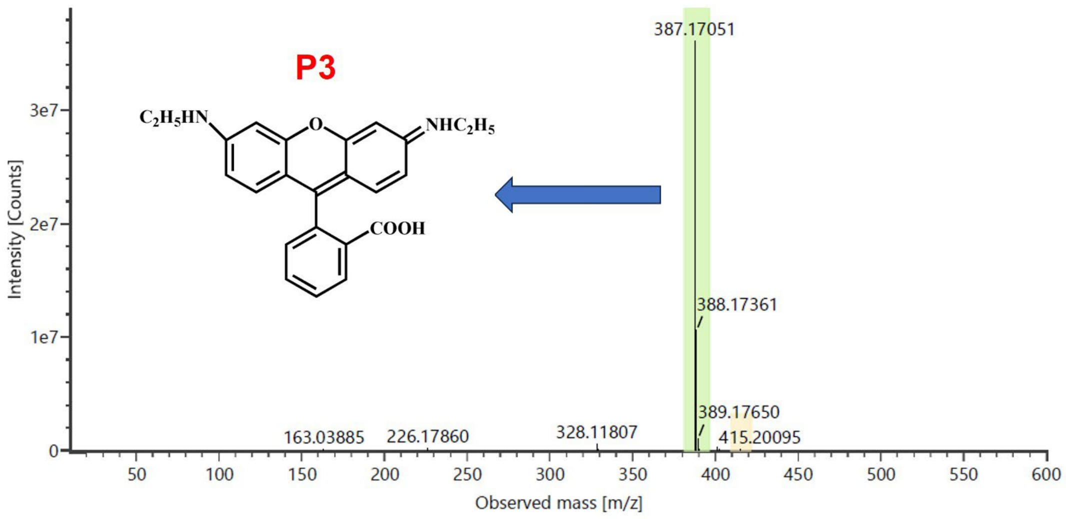
P3
387.17051
388.17361
389.17650
163.03885
226.17860
328.11807
415.20095
Intensity [Counts]
Observed mass [m/z]
COOH

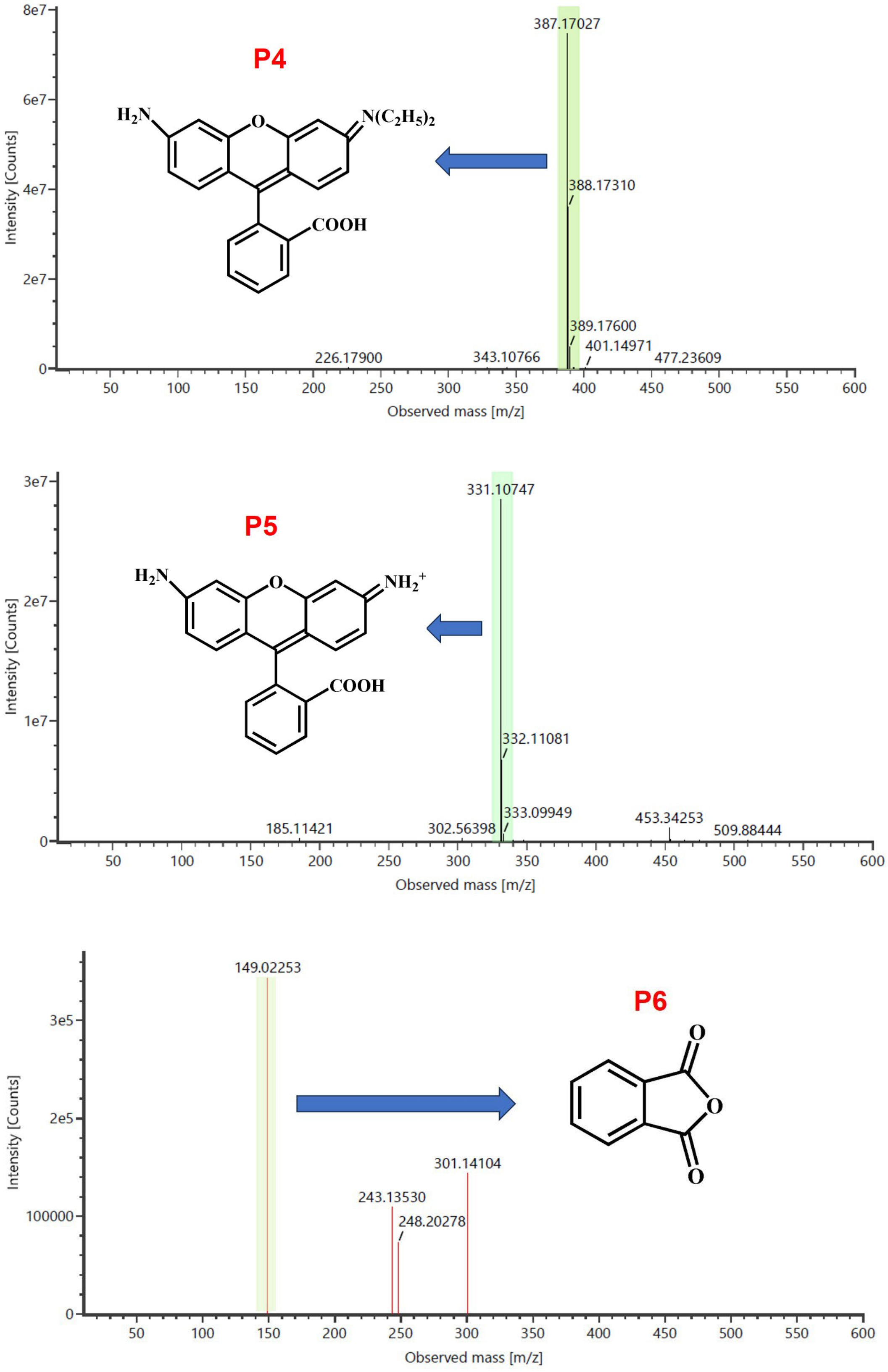

Figure S8. The possible intermediates of RhB degradation under visible light.

Table S1. Comparison of the photocatalytic performance of several composites for the degradation of pollutants.

Photocatalysts	Light sources	Pollutant	Degradation efficiency	Concentration (mg L^{-1})	Dosage (g L^{-1})	Kinetic constants	Refs.
Bi_2WO_6/g-C_3N_5	Tungsten lamp (300W)	TC	93.0 % (90 min)	10	0.6	0.0299 min^{-1}	[1]
CdTe/Bi_2WO_6	Xenon lamp (300 W)	TC	91.4 % (135 min)	20	0.5	0.0143 min^{-1}	[2]
$Cu_2(OH)_3F$/Bi_2WO_6	Xenon lamp (300 W)	TC	91.0 % (60 min)	20	0.2	0.0293 min^{-1}	[3]
CQDs/Bi_2WO_6	Xenon lamp (300 W)	TC	89.0 % (40 min)	20	0.6	0.0461 min^{-1}	[4]
CSs-Bi_2WO_6	Xenon lamp (300 W)	TC	84.6 % (60 min)	50	0.5	0.0333 min^{-1}	[5]
WS_2/Bi_2WO_6	Xenon lamp (350 W)	RhB	95.0 % (100 min)	10	0.4	0.0265 min^{-1}	[6]
P-$La_2Ti_2O_7$/Bi_2WO_6	Xenon lamp (300 W)	RhB	99.0 % (80 min)	10	1.0	0.0622 min^{-1}	[7]
In_2S_3/Bi_2WO_6	Xenon lamp (500 W)	RhB	93.0 % (30 min)	15	0.3	0.0416 min^{-1}	[8]
Sn/Bi_2WO_6	Xenon lamp (250 W)	MB	92.0 % (60 min)	10	0.25	0.0310 min^{-1}	[9]
$BiPO_4$/Bi_2WO_6	Xenon lamp (500 W)	MB	88.2 % (60 min)	10	0.5	0.0305 min^{-1}	[10]
**FeOOH/ZCS**	Xenon lamp (300 W)	TC	99 % (25 min)	50	0.25	0.174 min^{-1}	**This work**
**FeOOH/ZCS**	Xenon lamp (300 W)	RhB	98 % (40 min)	20	0.25	0.083 min^{-1}	**This work**
**FeOOH/ZCS**	Xenon lamp (300 W)	MB	95 % (25 min)	20	0.25	0.095 min^{-1}	**This work**

References

1. Rajendran, S.; Chellapandi, T.; UshaVipinachandran, V.; Venkata Ramanaiah, D.; Dalal, C.; Sonkar, S. K.; Madhumitha, G.; Bhunia, S. K., Sustainable 2D Bi_2WO_6/g-C_3N_5 heterostructure as visible light-triggered abatement of colorless endocrine disruptors in wastewater. *Applied*

Surface Science **2022,** 577.

2. Yang, P.; Chen, C.; Wang, D.; Ma, H.; Du, Y.; Cai, D.; Zhang, X.; Wu, Z., Kinetics, reaction pathways, and mechanism investigation for improved environmental remediation by 0D/3D CdTe/Bi_2WO_6 Z-scheme catalyst. *Applied Catalysis B: Environmental* **2021,** 285.
3. Wang, L.; Liu, Y.; Lin, Y.; Zhang, X.; Yu, Y.; Zhang, R., Z-scheme $Cu_2(OH)_3F$ nanosheets-decorated 3D Bi_2WO_6 heterojunction with an intimate hetero-surface contact through a hydrogen bond for enhanced photoinduced charge separation and transfer. *Chemical Engineering Journal* **2022,** 427.
4. Ren, H.; Qi, F.; Labidi, A.; Zhao, J.; Wang, H.; Xin, Y.; Luo, J.; Wang, C., Chemically bonded carbon quantum dots/Bi_2WO_6 S-scheme heterojunction for boosted photocatalytic antibiotic degradation: Interfacial engineering and mechanism insight. *Applied Catalysis B: Environmental* **2023,** 330, 122587.
5. Jiang, X.; Chen, S.; Zhang, X.; Qu, L.; Qi, H.; Wang, B.; Xu, B.; Huang, Z., Carbon-doped flower-like Bi_2WO_6 decorated carbon nanosphere nanocomposites with enhanced visible light photocatalytic degradation of tetracycline. *Advanced Composites and Hybrid Materials* **2023,** 6, (1), 47.
6. Su, M.; Sun, H.; Tian, Z.; Zhao, Z.; Li, P., Z-scheme 2D/2D WS_2/Bi_2WO_6 heterostructures with enhanced photocatalytic performance. *Applied Catalysis A: General* **2022,** 631, 118485.
7. Li, J.; Zhao, Y.; Xia, M.; An, H.; Bai, H.; Wei, J.; Yang, B.; Yang, G., Highly efficient charge transfer at 2D/2D layered P-$La_2Ti_2O_7$/Bi_2WO_6 contact heterojunctions for upgraded visible-light-driven photocatalysis. *Applied Catalysis B: Environmental* **2020,** 261, 118244.
8. Salunkhe, T. T.; Gurugubelli, T. R.; Babu, B.; Yoo, K., Recent Innovative Progress of Metal Oxide Quantum-Dot-Integrated g-C3N4 (0D-2D) Synergistic Nanocomposites for Photocatalytic Applications. *Catalysts* **2023,** 13, (11).
9. Zhu, X.; Qin, F.; Zhang, X.; Zhong, Y.; Wang, J.; Jiao, Y.; Luo, Y.; Feng, W., Synthesis of Tin-Doped Three-Dimensional Flower-like Bismuth Tungstate with Enhanced Photocatalytic Activity. *International Journal of Molecular Sciences* **2022,** 23, (15), 8422.
10. Zhu, Y.; Wang, Y.; Ling, Q.; Zhu, Y., Enhancement of full-spectrum photocatalytic activity over $BiPO_4$/Bi_2WO_6 composites. *Applied Catalysis B: Environmental* **2017,** 200, 222-229.
